# An IoT Smart Rodent Bait Station System Utilizing Computer Vision

**DOI:** 10.3390/s20174670

**Published:** 2020-08-19

**Authors:** Robert Ross, Lyle Parsons, Ba Son Thai, Richard Hall, Meha Kaushik

**Affiliations:** 1 Department of Engineering, La Trobe University, Melbourne, VIC 8083, Australia; l.parsons@latrobe.edu.au (L.P.); T.Thai@latrobe.edu.au (B.S.T.); R.Hall@latrobe.edu.au (R.H.); 2 International Institute of Information Technology (IIT) Hyderabad, Hyderabad 411057, India; Meha.Kaushik@Microsoft.com

**Keywords:** Wireless Sensor System, machine vision, Internet of Things, pest management, Remote Sensing

## Abstract

Across the world billions of dollars of damage are attributed to rodents, resulting in them being classified collectively as the biggest animal pest in the world. At a commercial scale most pest control companies employ the labour intensive approach of deploying and manually monitoring rodenticide bait stations. In this paper was present, *RatSpy*, a visual, low-power bait station monitoring system which wirelessly reports both on bait station levels and intruders entering the bait station. The smart bait stations report data back to a custom designed cloud platform. The system performance was evaluated under realistic field conditions (on an active cattle farm) with initial results showing significant potential in terms of reducing manual labour, improving scalability and data.

## 1. Introduction

Commensal rats, particularly the brown or Norway rat (*Rattus norvegicus*) and black or roof rat (*Rattus rattus*), cause major problems around the world in relation to *disease*, *damage* and *food competition*. Regarding *disease*, rats can assist in the transmission of diseases including dysentery, bubonic plague and typhus fever. The black rat is thought to have played an instrumental role in the spread of the Great Plague of London during the 17th century which killed approximately 25% of London’s population as well as 10’s of millions across Europe [1].

In terms of *property damage*, rats leave droppings, hair and gnaw which commonly damages carpets and furniture [2]. More seriously, rats can damage sensitive electrical equipment and even cause fires by gnawing through electrical wires [3].

*Food competition* becomes a significant problem as rats and mice are fast breeders with populations of rats estimated at 250 million in U.S. suburban and urban areas and up to 1 billion on poultry farms resulting in a loss of billions of dollars annually from contamination and consumption of food [3]. In total rats are estimated to cause well over $19 billion of damage annually. These costs make rats the most destructive animal pest and not that far behind the most destructive pests (in the U.S.), namely: crop weeds and crop pathogens at damage bills of $24 billion and $21 billion respectively.

Consequently, significant effort has gone into the development of rodenticide [4,5]. The most widespread rodenticides in common use are anticoagulants which interfere with the action of vitamin K, preventing clotting and causing animals to die from internal bleeding over the course of several days (typically outside of the bait station) [6,7]. Second-generation poisons, like brodifacoum and bromadiolone, are more potent and will kill rodents after a single feeding. In a given population some rodents may have a resistance to the poison or display significant neophobia which will keep them away from bait stations [8].

Businesses often contract out their pest control to specialist pest management companies to mitigate risk and ensure professional management of pests. Despite widespread rodent impact, rat extermination is becoming increasingly regulated for two main reasons. Firstly, according to records of poison control reports, thousands of children are feared to ingest some amount of rat poison annually year with 80% of reported exposures for children below the age of 6 [9]. Secondly, rat poison doesn’t just kill rats. Aside from killing other animals that eat the poison, including pets, it subsequently kills many wild animals further up the food chain, who eat the animals who have eaten the poison [10]. Further development is ongoing into specifies selective rodenticides (Norbormide) [11].

For these reasons, regulations around the world, particularly across Europe and the US, are moving towards requiring evidence of rat infestation to maximise the effectiveness of deployment of anticoagulant baits [12,13]. The European Committee for Standardisation (CEN) and the Confederation of European Pest Management Associations (CEPA) are currently defining standards to be adopted by pest control technicians. Current guidelines and best practice require detailed site and risk assessments, reduction of permanent toxic baiting, frequent inspections (at least fortnightly) [13]. These integrated approaches often incorporate: inspections, sanitation (removing food sources), rodent proofing (exclusion), population reduction (trapping or poisoning).

Hence, there is an increasing need to track and understand rat populations. Traditional tracking techniques currently include live trapping, scat analysis, chewing of oil cards or non-toxic wax baits and footprint tracking on track boards [14,15]. Poisoning is thus being positioned at the upper end of the hierarchy of control as a reactive measure [6].

One significant expense for these pest control companies is the servicing of bait stations which for larger premises may amount to several hundred stations [2]. The vast majority of these traps provide little or no back-to-base feedback and hence require repetitive and time-consuming maintenance to ensure that sufficient bait remains in the traps and that there is evidence of possible infestations to continue with toxic baiting strategies.

**Figure 1:**
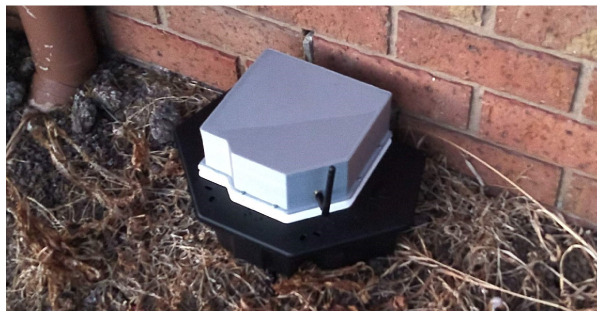
RatSpy fitted to a typical bait station.

This paper presents RatSpy—a low-powered, machine vision based wireless sensor package to be deployed in each bait station (shown in Figure 1). These sensor packages capture images triggered on defined time intervals (for bait estimation) and motion (for pest identification) to provide a unique insight to allow pest control experts to understand and respond to pest infestations. Our approach reduces the need for manual servicing (to check if bait remains) whilst provides timely information of what is happening out in the field.

This paper is structured as follows. Section 2 provides a review of related research and is followed by the system requirements for the proposed system in Section 3. Subsequently, its design and results are described in Section 4 and Section 5 respectively. Future work and concluding remarks are then provided in Section 6.

## 2. Related Work

Given the scale of the problem of rodent pest control a range of technological solutions have recently been formulated both to facilitate non-poisonous lethality and for in-field monitoring [16].

Non-rodenticide lethal traps current options now include CO_2_ poisoning, electrocution, strangulation, traditional mouse traps and self-resetting striking pistons [17,18,19,20]. Such systems have the advantage that no poison enters the food chain. They still require rats to enter the traps, offer little connectivity and require regular servicing which may make them unsuitable for many commercial environments as they leave a pile of dead rats [21].

There is significant increasing trend for systems which capture, process and transmit data, both for rodent pest control and pest control more generally. These include systems to track bait station entries (e.g., SmartEye), to allow entry when a rat is suspected (AutoGate) and relatively crude displacement sensors for estimating bait levels (RatTrace) [16,22,23]. Though these systems detect an intruder they provide sparse inferred information on what actually enters the bait station and consequently the condition of the bait station. Recent Internet of Things (IoT) applications have shown promise for rodent detection through an environment using motion sensors, background subtraction and edge detection [24,25]. Outside of rodent pest control IoT systems are finding significant in-roads into data driven pest management. These include visual systems (Trapview and GUPSY) and bio-impedance systems (Spensa Z-Trap) targeted towards insect pests [16]. A wild-pig selective trap was recently trialled and was found effective in deterring wild deer but less effective for raccoons [26].

In considering communications technologies within IoT based pest control applications three significant factors to consider are transmission distance, bandwidth and power. Specifically lower data size applications (e.g., where the presence of an intruder is logged) tend towards low-bandwidth communications like LoRaWAN which also have longer transmission distances with lower power requirements. Conversely, higher data size requirements (e.g., transmitting images) tend to use higher bandwidth and higher power communications (WiFi, NB-IoT, 4G/5G) [16].

## 3. System Requirements

The requirements for our system are summarized in Table 1 and Table 2. These tables are derived from the international standard ISO/IEC 25010:2011 model [27]. This model consists of two components which can be used to assess the quality of a system. The product quality model (Table 1) highlights various static and dynamic properties of the system, while the quality in use model (Table 2) gives characteristics related to user interactions with the system under specific contexts of use. We note that while this international standard is used primarily for evaluating software products, its characteristics are directly applicable to systems involving both hardware and software components.

## 4. System Design

**Figure 2:**
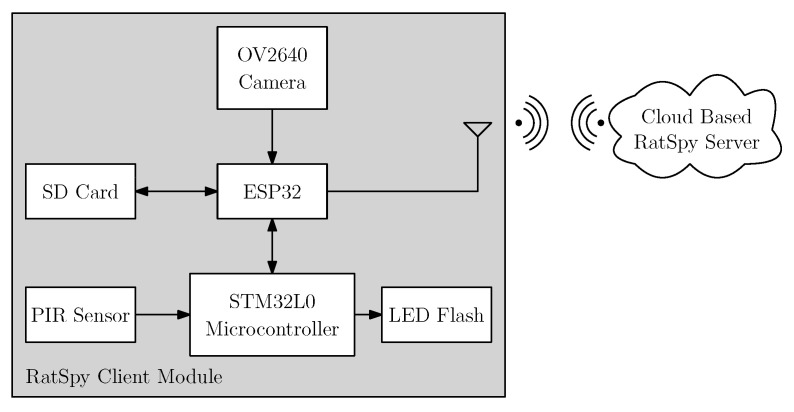
System Block Diagram.

**Figure 3:**
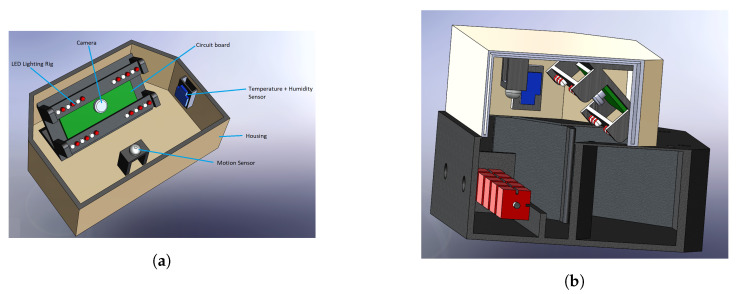
View of the Ratspy Client Module (**a**) and a cross-sectional view (**b**).

Figure 2 shows the block diagram of the entire system. The two main components of this system are the IoT node (Ratspy Client Module) and the cloud server (Ratspy Server). The Ratspy Client Module is mounted as a bolt-on to traditional bait stations. It consists of various electronic components which send information regarding the bait station to the Ratspy Server Module via a gateway device. Figure 3 illustrate how the Ratspy Client module is integrated into a traditional bait station. The individual components of the Ratspy Client Module are labelled in Figure 3a. As shown in Figure 3b, a cut-out must be made on the bait station prior to integrating it with the module. This enables the camera on the module to ‘see’ the inside of the inside of the bait station.

The client module is powered by two 1.5V AA batteries, which are mounted inside the RatSpy enclosure. An SD card, present on the client module stores information such as the WiFi SSID and password, the device token, captured images, sensor data, as well as some configuration parameters. The STM32L0 microcontroller block shown in Figure 2 is used to collect sensor data, as well as to selectively power the peripheral components surrounding it. The camera and SD card communicate directly with the ESP32 chip due to the memory constraints of the STM microcontroller.

**Figure 4:**
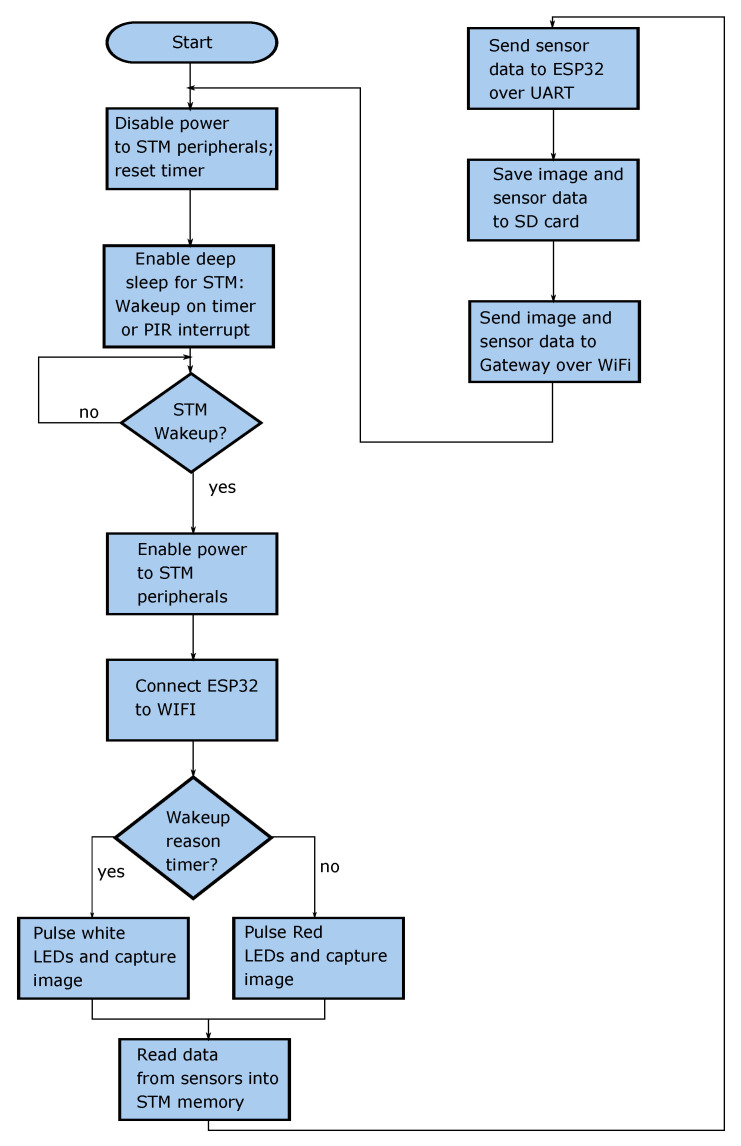
Flowchart for Ratspy Client Module.

The flowchart for the client module functionality is shown in Figure 4. The STM remains in deep sleep mode until either motion is detected in the bait station, or the timer interrupt occurs. The purpose of the timer interrupt is to periodically capture images of the bait. These images are sent to the cloud based server where they are analysed to determine the level of bait remaining as well as the type of bait used. By default, the timer interrupt will occur once every 24 h, however the duration between timer interrupts can be changed by modifying a configuration file on the SD card. When the timer interrupt occurs, the STM will turn on the ESP32 and camera module. The image is captured under white light provided by the LED lighting rig, and subsequently transferred to the RAM of the ESP32. The STM also turns on the sensors and acquires readings from them. A local copy of the image is then stored in the SD card, together with the sensor readings. Following this, an HTTP request message is generated by the ESP32, which contains the following information:

- Device token
- Temperature
- Humidity
- Battery Voltage
- Captured Image

The HTTP request is then sent from the ESP32 to the server module via the WiFi gateway device. The STM then returns to a deep sleep state, and disables power to its peripheral devices (except for the PIR sensor) so as to prolong the battery life. If the PIR sensor detects motion while the STM is in deep sleep mode, the client module will perform a similar sequence of tasks as mentioned above, however the image will be captured under red light instead of white light.

When the server recieves a HTTP request from a RatSpy client the device token is first checked against tokens of registered devices in the server database and the HTTP request is rejected if invalid. The device token acts as both a security measure as well as a unique identifier for each individual bait station. After the token has been validated, the captured image and sensor data are saved in the server database along with a time-stamp. Image processing is then performed on the captured image to determine the bait type and estimate the bait level. If the bait level or battery voltage is below a set threshold this is flagged on the website for service technicians to schedule site visits for maintenance. This is in contrast to the traditional pest management strategy, where site visits are periodically scheduled without prior knowledge of bait station activity. Hence, labour costs can be significantly reduced with the use of the RatSpy system. Information regarding all registered bait stations is presented on a website through various views such as heat maps as further described in the *Data Presentation* section of this paper.

### 4.1. Image Acquisition

**Figure 5:**
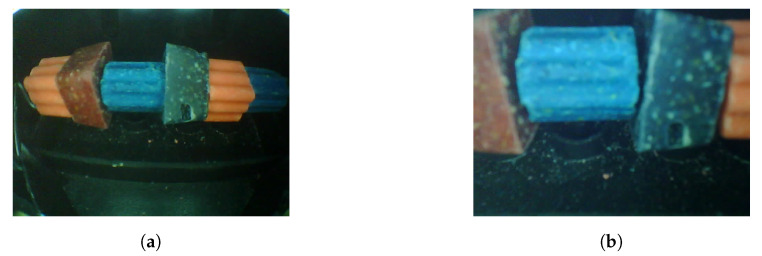
Captured images of the bait rod using a Fisheye lens (**a**), and without the Fisheye lens (**b**).

Placing a camera in the bait station in a position which captures the entire bait rod required the use of a wide angle lens. This is because the field of view of a typical small camera module is not wide enough to capture the entire area containing bait without moving it an unreasonable distance away. Adding a wide angle lens significantly increases the field of view, with the drawback of distortions being introduced into the captured image. The non-rectilinear design of the lens coupled with its short focal length allows for the camera to capture more of the real world (up to a 180° field of view) [28]. To illustrate the necessity for a wide angle lens in this system, Figure 5a,b show images captured by a camera placed 95 mm away from the bait rod with and without a lens respectively. It is evident that the full bait rod is visible in the image captured using the lens. The distortions are also visible in Figure 5a, where the horizontal edges of the bait appear as curved edges. This distortion is known as the Fish-eye effect and can be corrected using vision algorithms applied to the captured image [29].

Rodent bait stations are designed to eliminate as much ambient light as possible, so as to create a secluded environment in which the rodents can enter and consume the bait. To capture an image of the inside of the bait station, artificial lighting was therefore needed. Three factors were considered when designing the lighting system:

- To illuminate the entire bait rod so that the bait estimation algorithms worked optimally.
- To create an illumination system which would not scare a rodent away as soon as it is turned on.
- To optimize the lighting system so that it consumes low power when on.

There are two conditions when the lighting system should be illuminated. The first condition is when an image is captured for bait estimation. The second condition is when a rodent triggers the motion sensor and an image is captured for identification of rodent activity in the trap. White light was used to capture an image for bait estimation and red light was used to capture an image for rodent activity identification. The use of red light stems from the fact that rodents cannot see red light [30]. Hence illuminating the bait station with red light after the motion sensor is triggered will not cause the rodents to be startled.

**Figure 6:**
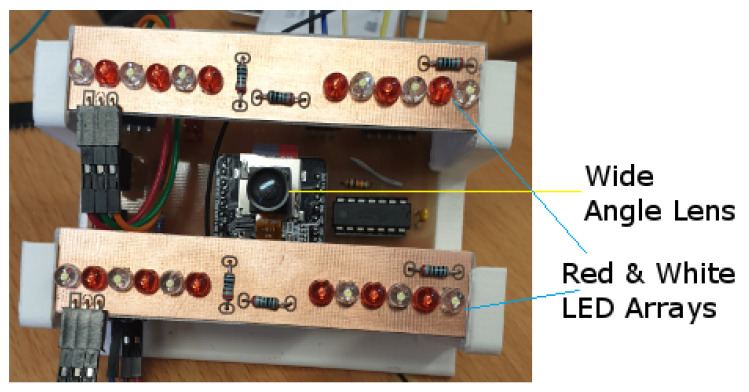
Artificial lighting for image illumination.

To produce even lighting while optimizing power, wide angle LEDs were used for illumination. The wide angle beam of each LED illuminates more of the bait rod than a narrow angle LED would thereby reducing the number of LEDs required along with the power consumed. A total of 12 white and 12 red LEDS were set up as shown in Figure 6 to produce the artificial lighting rig.

A small and inexpensive 2 mega pixel camera (OV2640) was used for image capture. The OV2640 communicates directly with the ESP32 module, transferring the captured image to the RAM of the ESP32. The camera is configured to output captured images in a compressed (JPEG) format, which are downsampled, reducing the need for compression to be implemented on the microcontroller. These more compact images reduces the amount of data sent over the WiFi network in comparison to using uncompressed bitmap images, but at between 20 K–40 K they are still orders of magnitude too larger to be sent using lower bandwidth communications IoT interfaces like LoRA. For the purpose of our bait estimation and intruder detection image processing algorithms, the loss in image quality due to the compressed JPEG format does not negatively affect our results.

### 4.2. Sensor Data Acquisition

Table 3 shows the types of sensors used in the RatSpy module, as well as the current consumed by each sensing device. While the STM microcontroller is in deep sleep mode, the Passive Infra-red (PIR) motion sensor remains powered while all other sensors are effectively off. Hence, using a PIR sensor with a low sleep current is required. The HC-SR501 PIR module has a small form factor and low sleep current. It is also a low cost device, making it ideal for this application. It was placed in line with the bait rod in order to detect motion within this region. Any motion sensed by this device wakes the STM32 micro-controller from its deep sleep mode, thereby initiating image capture and temperature and humidity measurement.

The purpose of measuring temperature and humidity is to monitor the environmental conditions in the bait station. The humidity level in a bait station can give an indication of how long unconsumed bait will last before it develops mould. Rodent activity can also be influenced by ambient temperature. They are more active (and hence more likely to search for food) in low to moderate temperatures [31]. The DHT11 sensor was chosen as it is a low cost sensor capable of measuring both temperature and humidity with an acceptable accuracy. It was placed along the internal wall of the bait station so as to measure ambient conditions within the bait station.

A resistive potential divider was used to measure the voltage of the batteries. The divider consists of two 100 K resistors in series. By remotely monitoring the battery voltage, technicians can be notified in advance of RatSpy modules requiring battery replacement.

### 4.3. Communications Infrastructure

**Figure 7:**
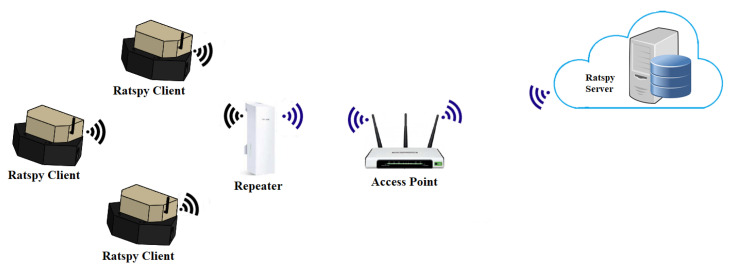
Ratspy Communications Infrastructure.

Figure 7 shows the communications infrastructure used for sending data from the RatSpy Client Module to the RatSpy Server Module.

Data is sent from the ESP32 WiFi module in the RatSpy Client to a long-range TP-LINK CPE220 Outdoor device, configured as a WiFi repeater. This device was chosen as it has a 12 dBi dual polarized directional antenna, making it capable of long distance communication of up to 13 km (as a point to point configuration with two matched devices). The ESP32 WiFI module uses an external omnidirectional antenna. Each client module connects to the WiFi provided by the access point through the SSID and password saved on the SD card configuration file. The access point requires an internet connection which could be provided from an existing wired connection or through a wireless connection (e.g., 4G SIM-Card). From a cost point of view this architecture is preferable compared to having a SIM card (e.g., 4G or NB-IoT) in every device. The configuration file also contains a static IP address for the client module which reduces communication time (saving power) as a DHCP request is not required. Once connected to the internet, the client modules can then send HTTP requests containing the data to be uploaded to the RatSpy Server Module. For the purpose of prolonging battery life, communication between the client and server is always initiated by the client module. The server sends an acknowledgement of received data in the form of an HTTP response to the client module.

### 4.4. Image Processing

Image processing is performed on the server with scripts written in Python leveraging the OpenCV computer vision library. The scripts run each time an image is saved to the database, and the results of the image processing are saved to the database and presented to the user via the website. The image processing details are further documented in [32] and demonstrate the efficacy of colour segmentation, background subtraction and edge detection. At a high level the fundamental requirements were:

- Identify the type of bait in the image
- Estimate the bait level in the image
- Detect whether there is a rodent in the image

### 4.5. Data Presentation

Information from the bait stations can be viewed by logging into a custom designed website. The website has user profiles and appropriate security which allows users to be segmented based on the bait stations registered to their account. This allows for unique alerts to be setup (e.g., email or reports) based on bait stations or clusters of bait stations which require servicing.

After successful login, a user can view statistics of each registered bait station in their profile, as well as view images captured by those bait stations. The bait levels are also superimposed on a map, with pins representing the location of each bait station. Users can also register new bait stations on the website. During registration, a unique device token is generated. This token is saved to the configuration file on the SD card of the corresponding RatSpy Client Module.

### 4.6. Power Considerations

Battery longevity is essential for an IoT system such as this as we don’t wish to simply supplant bait restocking with battery restocking. Low-power, low-bandwidth communications like LoRaWAN and NB-IoT unfortunately don’t satisfy the bandwidth requirements for sending images as required by the RatSpy module. A typical HTTP request from a RatSpy Client Module would exceed the daily maximum limits for most LPWAN technologies.

3G/4G modules could be used but these consume significant power and with a SIM card per bait station would significantly increase cost. Hence we chose long-range WiFi as our communications backbone as it is relatively low power, has sufficient bandwidth and is low cost.

One of the requirements listed within the product quality model was for a long battery life (6 months). To achieve this both hardware and software design decisions were optimised.

In terms of hardware approaches, low power electronic components were chosen. For example, the LM3671 switching regulator was chosen to provide a regulated supply voltage to the system as it has a typical quiescent current of only 16 μA. This current is much lower than that of the AMS1117 regulator used on most ESP32 breakout boards, which has a quiescent current of 5 mA. The STM32L0 microcontroller was chosen from the ultra low power family of STM microcontrollers with a current consumption as low as 230 nA in deep sleep mode. To further reduce the overall power consumption of the system, the STM microcontroller uses MOSFETS to switch off peripheral devices (the ESP32, the camera, the SD card, the temperature and humidity sensor, and the LED lighting rig) when they are not being used.

In terms of software approaches for reducing the system’s overall power consumption, the following methods were implemented:

- Placing the STM microcontroller into deep sleep when no motion is detected in the trap and no timer interrupt is triggered.
- Limiting the number of images captured for bait estimation to one per day if the bait station is visited often, or one per week if the bait station is seldom visited.
- Limiting the number of images captured while an intruder is present in the bait station, regardless of how long the intruder stays in the station.
- Limiting the number of times the RatSpy Client Module connects to the RatSpy Server Module. Captured images can be saved to the SD card and later sent as a batch at a specified time (for example once every few days, depending on the number of images captured).

By combining the above mentioned hardware and software approaches, it is possible to significantly reduce the power consumption of the RatSpy Client Module, thereby reducing the frequency of site visits for battery replacement. Lithium batteries were chosen for this system as they have very low leakage current, have better temperature stability and have larger capacity than their alkaline AA counterparts [33]. For even longer battery life, the system can be powered with C or D batteries.

## 5. System Evaluation and Discussion

In this section we evaluate the system performance of the RatSpy system in terms of image clarity, bait estimation, triggering and power efficiency. Table 4 compares RatSpy with traditional and state of the art approaches to rodenticide based rodent control systems. Here we contrast 4 different approaches:

- Traditional: Bait checked manually by human operators every 2 weeks. Rodent scat observed and noted in the field
- Trigger Report: A PIR sensor or break-beam sensor is used to record presence of an intruder which is logged to a server
- Trail Camera: Motion triggered cameras capture images of animals moving nearby
- Displacement Sensor: A high-resolution rangefinder for estimating bait levels
- Vision Estimation and Capture (RatSpy): Using machine vision to estimate bait levels and capture pictures of intruders

### 5.1. Image Clarity

**Figure 8:**
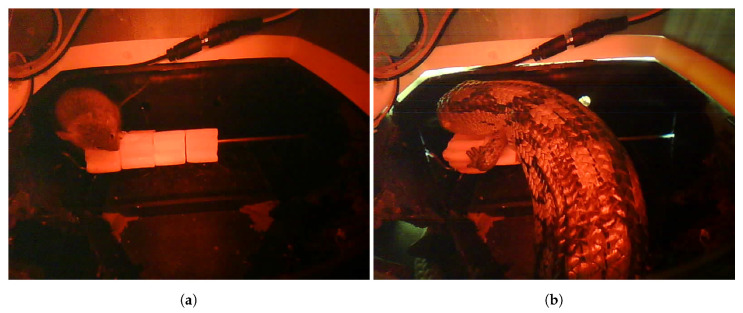
Motion triggered captures showing a rat (**a**) and a blue-tounge lizard (**b**) from a farm in rural Victoria.

**Figure 9:**
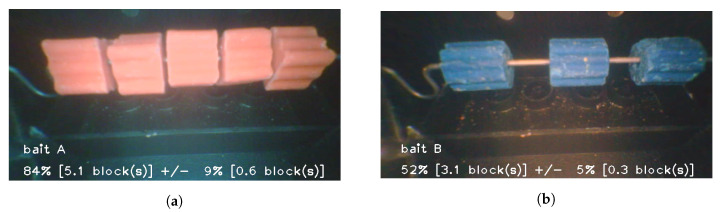
Image processing outputs for bait identification and bait level estimation. (**a**) Five blocks of red bait detected; (**b**) Three blocks of green bait detected.

Images are captured for pest classification with red light (Figure 8) and for bait estimation with white light (Figure 9). The white images (as discussed later) provide sufficient clarity to perform bait type classification (between red, blue, green and pasta bait) and bait estimation. Visually the bait images also allow bait condition to be inspected (for mould), a process we expect to automate into the future.

Expert zoologists at our commercial partners has advised us the image clarity for the red pest images is good and allows them to both to manually identify visitors and observe what they are actually doing within the bait station. These pest images also confirm that other animals besides rodents frequent the bait stations—to date we have also observed lizards and spiders.

### 5.2. Bait Estimation

Figure 9 shows the output of the image processing bait estimation algorithms. In each image shown in the figure, there is a bait type (A-E), as well as the bait level which is accurate to a tolerance specific to the bait type. The tolerance was experimentally derived based on the bait shape (as the larger square blocks tended to produce more occulusions). A background subtraction algorithm coupled with a colour segmentation algorithm was used to automatically provide an estimate for bait quantity and type.

This bait estimation approach gives a significant advantage on the state of the art as it allows directly measures bait levels rather than relying on regular checking or inferring bait levels based on trap activity.

### 5.3. Triggering Efficacy

Image capture is triggered via two separate means. Firstly, periodic timer driven capture for bait estimation reliably operates on an interval assigned by the user. Secondly, a passive infrared (PIR) detector is used as a motion detector to trigger the camera to the presence of a visitor. The time to capture an image from when the PIR triggers a capture event is 1600 ms with a further 2500 ms required to upload the image to the cloud server. Table 5 summarises what was observed to trigger the PIR capture over the course of two months of monitoring.

The spider had a disproportionately high number of triggers as it decided to take up residence in the bait station (the spider and associated webs would be cleared away on the next trap service interval). A significant portion of the images have an unknown trigger source. We postulate that this could have been a small insect that we didn’t notice in the image or something that was within the field of view of the PIR sensor but was not seen by the camera. Given the significant number of insect and unknown triggerings, image based filtering (removing images that haven’t been triggered by larger animals) or different sensors (e.g., a break-beam or capacitive proximity sensor) may minimise these unwanted images.

Interestingly no rodents were detected during the first two weeks after the bait station was installed. This observation matched the long understood neophobia exhibited by wild rats [35].

### 5.4. Power Efficiency

Table 6 aggregates the power consumption for each of the elements within the RatSpy device given their fractional usage time assuming 4 images per day are captured. Given that the AA lithium batteries have a capacity rating of approximately 3000 mAh, this will provide approximately 6 months of battery life. Alternatively, a single 18,650 rechargeable LiPO cell may be used and swapped over at 6 month intervals. Given that battery voltage is periodically logged and that periodically bait will require refill, the battery changes would ideally made to coincide with these service visits to reduce servicing costs.

## 6. Conclusions

In this paper we present RatSpy—a prototype system facilitating low-cost visual monitoring inside rodent bait stations. RatSpy offers two significant advantages over traditional approaches and the current state of the art for rodent pest control. Firstly, it allows remote monitoring of rodenticide levels using a machine vision algorithm which alerts operators when refilling is required. Secondly, RatSpy captures images of intruders visiting the trap which is important to rapidly react to infestations and enables pest control operators to demonstrate evidence of infestation to relevant environmental authorities to support the use of toxic rodenticide. Hence the system provides a significant labour advantage whilst providing timely data from what is actually going on in the field.

In terms of improvements we see several areas which would improve system performance. Firstly, the system currently does not perform intruder classification. This classification, using for example a convolutional neural network, would allow clusters of pest or non-pest intruders to be quickly and automatically detected. Although this classification could be performed on the edge, we feel that cloud based classification is more appropriate given the increased processing power and decreased battery life if implemented on the edge. Small increases in latency for a cloud based classification system are offset by the fact that real-time notifications are not required as pest control companies rely on the rodenticide rather than live trapping. One significant possible advantage of classification on the edge would be to create a bait station which is only accessible to target species based on machine learning approaches. Secondly, our triggering system (currently a PIR detector) shows mixed results with small insects triggering the system. To solve this a more robust (e.g., break-beam or capacitive proximity sensor) system could be used as a trigger. Finally, we expect that the system could be enhanced to automatically detect bait spoilage (mould which develops at different rates depending on the environment) as this can currently be remotely manually performed using the captured images.

We finally consider the extent to which our design met our desired requirements. In terms of evaluating *product quality*, our system rapidly uploads images to the cloud triggered by both timer events (daily bait readings) and intruders. Cloud based visualisation for these images is implemented both as a numerical bait estimate and an image which can be used to verify bait estimates and observe intruders. Hence, this approach efficiently enables wide-scale monitoring of collections of bait stations and allows a faster response time to rodent infestations. The visual based approach provides a rich source of information to reduce risk and unintended environmental impact.

In terms of *Quality in Use* we show scope to significantly increase both efficiency and effectiveness in bait station management. The system maintenance is relatively low only requiring battery replacement at 6 month intervals which is double to interval required for bait to be replenished to avoid bait spoilage. Further field testing in partnership with an commercial pest control entity at a commercial-scale are required in a variety of different target environments is required to fully satisfy the requirements related to trust and usefulness.

## Figures and Tables

**Table 1:** Product Quality Model.

Characteristic	Sub-Characteristic	Requirements
Functional Suitability	Functional Completeness	Upload image for analysis of bait type, bait level, and intruder occurrences. Upload temperature, humidity, and battery level data
	Functional Correctness	Bait level estimate within 15%. Temperature accurate to ±2 °C and humidity to 5%
	Functional Appropriateness	Integration with existing bait station hardware
Performance Efficiency	Time-behaviour	Bait images captured once per day. Intruder images captured each time PIR sensor is triggered
	Resource Utilization	Inexpensive system which reduces labour costs involved with manual bait station inspections
	Capacity	Can operate for up to 6 months from AA batteries
Compatibility	Co-existence	System can be used with traditional bait stations
	Interoperability	Connects to any WiFi network (WPA security). Bait stations act as clients to a cloud based server.
Usability	Appropriateness recognisability	Users see images of bait station intruders as well as bait levels
	Learnability	Simple to set up on site and register device on website
	Operability	User friendly website which displays on mobile and PC
	Accessibility	Service panel for battery replacement and maintenance
Reliability	Fault Tolerance	Data sent over WiFi is logged onto SD card for redundancy
	Recoverability	Manual reset button. Low battery warning sent to website. Critical data stored on SD card. Self-reset on battery replacement
Security	Non-Repudiation	Images from bait station are time and date stamped. Software tokens give each registered bait station a unique identifier
Maintainability	Reusability	New devices can be registered on website for monitoring
	Testability	Bait estimate confirmed against visual inspection. Intruder verified by uploaded images
Portability	Installability	WiFi initialization and token registration using data stored on SD card
	Replaceability	Add-on device can be field swapped

**Table 2:** Quality in Use Model.

Characteristic	Sub-Characteristic	Requirements
	Effectiveness	Up to date data logged to cloud for bait type, bait estimate, intruders, temperature, humidity and battery level
	Efficiency	Labour cost related to bait station monitoring minimized
Satisfaction	Usefulness	Improves pest management approach with near real-time data. Facilitates remote monitoring of widespread bait stations
	Trust	Visual inspection of uploaded images validates bait level estimates
Freedom from Risk	Economic Risk mitigation	Low cost per unit for system set-up and maintenance
	Health and Safety Risk Mitigation	Used as an add-on to traditional lockable bait stations
	Environmental Risk Mitigation	Visual inspection of images showing intruders can be used as an early indicator of non-target species consuming the bait
Context Coverage	Context Completeness	Preliminary field testing
	Flexibility	Can be modified for remote monitoring of other pest traps

**Table 3:** Sensors used in the Ratspy Module.

Sensing Device	Sensed Parameter	Accuracy	Current Consumed
DHT11	Temperature + Humidity	±2 °C and ±5% respectively	2.5 mA
HC-SR501 PIR	Motion	-	450 μA (sleep), 8.9 mA (active)
100 K Voltage Divider	Battery Voltage	±1%	15 μA at 3 V
OV2640 2MP camera	Visual (Image)	-	40 mA (active)

**Table 4:** Comparison of RatSpy to alternate monitoring systems.

**Technology**	Traditional Baiting	Trigger Report	Trail Camera	Displacement Sensor	Vision Bait Estimation
**Examples**	Standard Practice [14]	SMARTeye, Pestconnect [17]	ScoutGuard [34]	RatTrace [23]	RatSpy
**Bait Level**	No—manual check	Inferred from number of intruders	No	Yes—Limited	Yes—automatic
**Bait Condition**	No—manual check	No—manual check	No—NA	No—manual check	Yes—manual from photo
**Visit Notification**	No	Yes	Yes	Yes	Yes
**Visitor Identification**	No—scat identification	No	Yes	No	Yes—manual from photo
**Remote Connectivity**	No	SIM	None or SIM	WiFi or LoRA	WiFi
**Data Latency**	2 week checking	<10 s	Manual or <10 s	<10 s	<10
**Additional Unit Cost (USD)**	Base	$50–$80	$50–$300	$20	$25

**Table 5:** Sensors used in the Ratspy Module.

Cause	Occurences
Rodent	15
Lizard	4
Spider	91
Unknown	55

**Table 6:** Power requirements for RatSpy components.

Mode	Average Current	Time Fraction
Sleeping	600 μA	99.984%
Image Capture	150 mA	0.004% (4 s per day)
Communication	200 mA	0.012% (10 s per day)
Average Current:	630 μA	
